# Effect of targeted therapy and immunotherapy on advanced nonsmall‐cell lung cancer outcomes in the real world

**DOI:** 10.1002/cam4.4427

**Published:** 2021-11-16

**Authors:** Aria Shokoohi, Zamzam Al‐Hashami, Sara Moore, Alexandra Pender, Selina K. Wong, Ying Wang, Bonnie Leung, Jonn Wu, Cheryl Ho

**Affiliations:** ^1^ Department of Medical Oncology BC Cancer Vancouver British Columbia Canada; ^2^ Faculty of Medicine University of British Columbia Vancouver British Columbia Canada; ^3^ Division of Medical Oncology The Ottawa Hospital Ottawa Ontario Canada; ^4^ Department of Radiation Oncology BC Cancer Vancouver British Columbia Canada

**Keywords:** chemotherapy, immunotherapy, medical oncology, nonsmall‐cell lung cancer, targeted therapy

## Abstract

The evolution of diagnosis and treatment of advanced nonsmall‐cell lung cancer (NSCLC) has led to increasing the use of targeted therapy and immune checkpoint inhibitors. The study goal was to assess the effect of molecular testing and the introduction of new therapies on overall survival (OS). All patients with stage IV NSCLC referred to BC Cancer were included in the study. Four 1‐year time cohorts were created based on molecular testing implementation and funded drug availability: C1 baseline (2009), C2 EGFR TKI access (2011), C3 ALK inhibitor access (2015), C4 immunotherapy availability (2017). Baseline demographics, disease characteristics, and systemic therapy details were collected retrospectively. OS was calculated using the Kaplan–Meier method and compared using the log‐rank test. There were 3421 patients identified with stage IV NSCLC and 1319 (39%) received systemic therapy. In the four 1‐year time cohorts C1/C2/C3/C4: driver mutation‐targeted treatment increased 1/17/27/34% (of total systemic therapy), as did treatment with any line immunotherapy <1/1/9/38%. Median OS with best supportive care (BSC) was 3.4/3.1/3.2/2.9 m (*p* = 0.16) and with systemic treatment 9.9/10.9/13.9/15.0 m (*p* < 0.001). Median OS by treatment exposure was BSC 3.1 m, chemotherapy only 7.3 m, targeted therapy 17.5 m, and immunotherapy 20.7 m. In our real‐world study, following the introduction of targeted therapy and immune checkpoint inhibitors, there was a significant improvement in OS in each successive time cohort concordant with advancements in therapeutic options.

## Graphical abstract

We evaluated outcomes with new therapies in 3421 patients with advanced nonsmall‐cell lung cancer. There was an improvement in survival over time with the introduction of EGFR, ALK, and immunotherapy. The median OS by treatment strategy was BSC 3.1 months, chemotherapy alone 9.2 months, driver mutation receiving targeted therapy 17.5 months, and immunotherapy in any line 20.2 months. Our findings in a real world demonstrate that it is critical to identify patients appropriately for emerging systemic therapies.

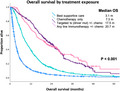

## 1 INTRODUCTION

Despite rapid changes and significant advancement in the past decade, lung cancer remains the leading cause of cancer‐related death worldwide with an estimated 1.8 million deaths each year.^1^ For decades, platinum‐based chemotherapy was the first‐line systemic treatment for advanced NSCLC. The landscape of NSCLC diagnosis and treatment has evolved, paving the way toward a more personalized approach to treatment. The introduction of targeted therapy and immunotherapy, whether alone or in combination with standard chemotherapy, has transformed the treatment paradigm.

The identification of driver mutations, such as epidermal growth factor receptor (EGFR), anaplastic lymphoma kinase (ALK), and c‐ros oncogene 1 (ROS1), and the associated targeted therapies have improved outcomes for NSCLC patients.^2^, ^3^, ^4^, ^5^ Additional actionable alterations including BRAF, MET, RET, HER2, and KRAS also offer opportunities for targeted treatments with less toxicity and potentially better outcomes than standard chemotherapy.^6^

The introduction of immune checkpoint inhibitors targeting programmed cell death protein 1 (PD‐1)/programmed cell death ligand 1 (PD‐L1), initially in the second‐line setting, and subsequently in the first‐line setting either alone or in combination with chemotherapy, has provided an additional therapeutic option for patients.^7^, ^8^, ^9^, ^10^, ^11^, ^12^ With the increasing treatment options, the proportion of patients who receive the first‐line platinum‐based treatment alone is diminishing.

The impact of the introduction of routine molecular testing, PD‐L1 evaluation, and associated therapies into the standard of care is presumed to result in survival benefits. Implementation in the real world, however, may not reflect the gains seen in clinical trials. Population‐based analyses allow for the evaluation of treatments outside the context of randomized controlled trials and assessment of whether these results are generalizable to real‐world patient populations.

The objectives of this retrospective study are to assess the effect of molecular testing and the introduction of new therapeutic options on the uptake of systemic therapy and to evaluate the change in overall survival (OS) for patients with advanced NSCLC in the real‐world setting.

## 2 METHODS

BC cancer is a provincial cancer care program that serves a population of 5.1 million residents in British Columbia (BC). Based on reporting to the Canadian Cancer Registry and the BC Cancer Surveillance and Outcomes Unit analysis, approximately 80% of advanced NSCLC patients are referred to the provincial program. BC Cancer is a single‐payer healthcare system and as a result has completed records on the billing and prescribing of all cancer therapies in BC. Treatment funding decisions are based on eligibility and exclusion criteria in a protocol defined by the tumor group context experts and vetted by the provincial systemic therapy program. Information regarding death dates is provided through linkage with Canadian Vital Statistics.

A retrospective chart review of all patients referred to BC Cancer with stage IV NSCLC was conducted using the Outcomes and Surveillance Integrated System (OaSIS) database and electronic medical records. Four 1‐year time cohorts were created to reflect the implementation of molecular testing and availability of funded drugs for NSCLC. Cohorts pre‐2010 (i.e., cohort 1) was staged using the American Joint Committee on Cancer staging manual version 6 and all subsequent cohorts used version 7.

Cohort 1 (C1) was the baseline cohort and included patients diagnosed from January to December 2009. In C1, there was no funded access to molecular testing and there were no provincially funded therapies associated with molecular aberrations.

Cohort 2 (C2) included patients diagnosed from January to December 2011, after the implementation of EGFR testing and provincial funding of first‐line gefitinib for EGFR mutation‐positive patients (October 2010).

Cohort 3 (C3) included patients diagnosed from January to December 2015, after the adoption of ALK testing and provincial funding of crizotinib (second line March 2014, first line December 2015).

Cohort 4 (C4) included patients diagnosed from January to December 2017, after implementation of PD‐L1 testing and the provincial funding of immune checkpoint inhibitors (second‐line nivolumab March 2017, second‐line pembrolizumab February 2018).

Data were collected on baseline demographic and clinical characteristics, including age, sex, smoking history, Eastern Group Cooperative Group (ECOG) performance status (PS), and histology. Systemic therapy details, including drug, type of treatment, number of cycles, and line of therapy, were collected. The immunotherapy group was defined as any treatment containing immune checkpoint inhibitors, including combinations with chemotherapy, and excluding patients with driver mutations treated with targeted therapy.

OS was calculated from the date of stage IV NSCLC diagnosis to date of death, with living subjects censored on the date of their last follow‐up. OS was estimated using the Kaplan–Meier method and compared with the log‐rank test. Univariate analyses were conducted using chi‐squared tests, Fisher's exact tests, and Kruskal–Wallis tests where appropriate. Multivariable analysis of OS was performed using the Cox regression model. All statistical analyses were conducted using IBM SPSS Statistics software, version 26 (IBM Corp), and statistical significance was set at a *p*‐value of <0.05.

This retrospective study received approval from the University of British Columbia BC Cancer Research Ethics Board; H15‐02509. A waiver of consent was obtained due to the minimal risk involved.

## 3 RESULTS

A total of 3421 patients with stage IV NSCLC were referred to BC Cancer in the four 1‐year time cohorts: 586 in C1; 803 in C2; 1,026 in C3; and 1,006 in C4. The median duration of follow‐up for the cohorts was 2009: 81.3 m, 2011: 68.04 m, 2015: 43.5 m, and 2017: 25.4 m. The data cutoff was August 30, 2019. In the whole study population, the median age at diagnosis was 69 years, 50% of patients were female, the most common histology was nonsquamous 58%, 55% presented with ECOG PS ≥2 at diagnosis, 82% were former/current smokers. The distribution of sex, the median age at diagnosis, histology, ECOG PS, and smoking history in the four cohorts is summarized in Table 1 (p value represents comparison over time cohorts).

**TABLE 1:** Baseline demographics and clinical characteristics of patients with nonsmall‐cell lung cancer by year of diagnosis

Baseline Characteristics	Whole population (*n* = 3421)	C1 2009 (*n *= 586)	C2 2011 (*n* = 803)	C3 2015 (*n* = 1026)	C4 2017 (*n* = 1006)	*p*‐value
Sex						
Female	1701 (50%)	279 (48%)	394 (49%)	532 (52%)	496 (49%)	0.374
Male	1720 (50%)	307 (52%)	409 (51%)	494 (48%)	510 (51%)	
Age at diagnosis, median (IQR), years	69 (61–66)	69 (60–76)	69 (61–76)	70 (62–78)	70 (62–78)	0.006
Histology						
Nonsquamous	1987 (58%)	225 (38%)	500 (62%)	639 (62%)	623 (62%)	<0.001
Squamous	484 (14%)	85 (15%)	123 (15%)	156 (15%)	120 (12%)	
NOS and other	950 (28%)	276 (47%)	180 (23%)	231 (23%)	263 (26%)	
ECOG at diagnosis						
PS 0–1	1101 (32%)	195 (33%)	293 (36%)	322 (31%)	291 (29%)	<0.001
PS ≥ 2	1888 (55%)	341 (58%)	391 (49%)	566 (55%)	590 (59%)	
Unknown	432 (13%)	50 (9%)	119 (15%)	138 (14%)	125 (12%)	
Smoking History						
Never	507 (15%)	69 (12%)	106 (13%)	173 (17%)	159 (16%)	<0.001
Former	1447 (42%)	245 (42%)	320 (40%)	436 (43%)	446 (44%)	
Current	1377 (40%)	267 (45%)	356 (44%)	381 (37%)	373 (37%)	
Unknown	90 (3%)	5 (1%)	21 (3%)	36 (3%)	28 (3%)	

Abbreviations: ECOG, Eastern Cooperative Oncology Group; IQR, interquartile range; NOS, not otherwise specified; PS, performance status.

Of the 3421 stage IV NSCLC patients referred for BC Cancer, 1319 (39%) patients were treated with systemic therapy, whereas the remaining 2102 (61%) received best supportive care (BSC). Of the patients who were treated with systemic therapy, 824 (62%) received chemotherapy alone, 304 (23%) were identified to have a driver mutation and received targeted therapy, and 191 (15%) received immunotherapy during the course of their disease.

Systemic therapy details, including the type of treatment, lines of therapy, and use of drugs in first‐ to fourth‐line systemic treatments are summarized in Table 2. Examination of first‐line treatment patterns by time cohorts demonstrated that rates of systemic therapy use over best supportive care remain stable with a trend toward more uptake from 2011 onward. The first‐line chemotherapy usage declined over the years with increasing delivery of targeted therapy and in the 2017 cohort, immunotherapy. The use of second‐line treatment or greater is higher in 2009 and 2011 compared with 2015 and 2017, respectively. This trend is paralleled by a diminishing use of second‐line EGFR tyrosine kinase inhibitors (TKIs) as it shifts to first‐line therapy.

**TABLE 2:** Systemic therapy details of patients with advanced nonsmall‐cell lung cancer by year

	C1 2009	C2 2011	C3 2015	C4 2017	*p*‐value
	*n* = 586	*n* = 803	*n* = 1026	*n* = 1006	
Treatment					0.095
Best supportive care	386 (66%)	482 (60%)	630 (61%)	604 (60%)	
Any systemic treatment	200 (34%)	321 (40%)	396 (39%)	402 (40%)	
Systemic treatment					<0.001
Chemotherapy only	196 (98%)	262 (82%)	254 (64%)	112 (28%)	
Driver mutation treated with targeted therapy ± chemotherapy	3 (1%)	55 (17%)	108 (27%)	138 (34%)	
Any line immunotherapy ± chemotherapy	1 (<1%)	4 (1%)	34 (9%)	152 (38%)	
First‐line treatment	200	321	396	402	<0.001
Platinum doublet	172 (86%)	254 (79%)	276 (70%)	219 (55%)	
Single agent	23 (11%)	17 (6%)	15 (4%)	4 (1%)	
EGFR TKI	2 (1%)	46 (14%)	83 (21%)	113 (28%)	
ALK inhibitor	0 (0%)	0 (0%)	18 (4%)	17 (4%)	
Immunotherapy	0 (0%)	0 (0%)	3 (1%)	44 (11%)	
Other	3 (2%)	4 (1%)	1 (<1%)	5 (1%)	
Second‐line treatment (% of patients who received prior line of tx)	115 (58%)	192 (60%)	145 (37%)	187 (46%)	<0.001
Platinum doublet	10 (9%)	31 (16%)	20 (14%)	25 (14%)	
Docetaxel	5 (4%)	2 (1%)	19 (13%)	1 (<1%)	
Pemetrexed	33 (29%)	49 (26%)	26 (18%)	3 (2%)	
EGFR TKI	67 (58%)	99 (52%)	39 (27%)	21 (11%)	
Osimertinib	0 (0%)	0 (0%)	11 (8%)	28 (15%)	
ALK inhibitor	0 (0%)	1 (<1%)	1 (1%)	1 (<1%)	
Immunotherapy	0 (0%)	2 (1%)	24 (16%)	103 (55%)	
Other	0 (0%)	8 (4%)	5 (3%)	5 (3%)	
Third‐line treatment (% of patients who received prior line of tx)	54 (47%)	89 (46%)	61 (42%)	67 (36%)	<0.001
Platinum doublet	3 (6%)	10 (11%)	4 (7%)	17 (25%)	
Docetaxel	2 (4%)	16 (18%)	8 (13%)	10 (15%)	
Pemetrexed	19 (35%)	23 (26%)	14 (23%)	7 (10%)	
EGFR TKI	25 (46%)	35 (40%)	16 (26%)	10 (15%)	
Osimertinib	0 (0%)	0 (0%)	2 (3%)	5 (8%)	
ALK inhibitor	0 (0%)	1 (1%)	0 (0%)	0 (0%)	
Immunotherapy	0 (0%)	1 (1%)	14 (23%)	15 (22%)	
Other	5 (9%)	3 (3%)	3 (5%)	3 (5%)	
Fourth‐line treatment (% of patients who received prior line of tx)	9 (17%)	28 (31%)	24 (39%)	26 (39%)	0.037
Platinum doublet	0 (0%)	6 (21%)	3 (12%)	1 (4%)	
Docetaxel	1 (11%)	4 (14%)	5 (21%)	5 (19%)	
Pemetrexed	2 (22%)	1 (4%)	3 (12%)	5 (19%)	
EGFR TKI	1 (11%)	8 (28%)	5 (21%)	3 (12%)	
Osimertinib	0 (0%)	2 (7%)	0 (0%)	4 (15%)	
ALK Inhibitor	0 (0%)	1 (4%)	0 (0%)	0 (0%)	
Immunotherapy	0 (0%)	1 (4%)	5 (21%)	7 (27%)	
Other	5 (56%)	5 (18%)	3 (12%)	1 (4%)	

Abbreviations: ALK, anaplastic lymphoma kinase; EGFR, epidermal growth factor receptor; TKI, tyrosine kinase inhibitor.

**FIGURE 1:**
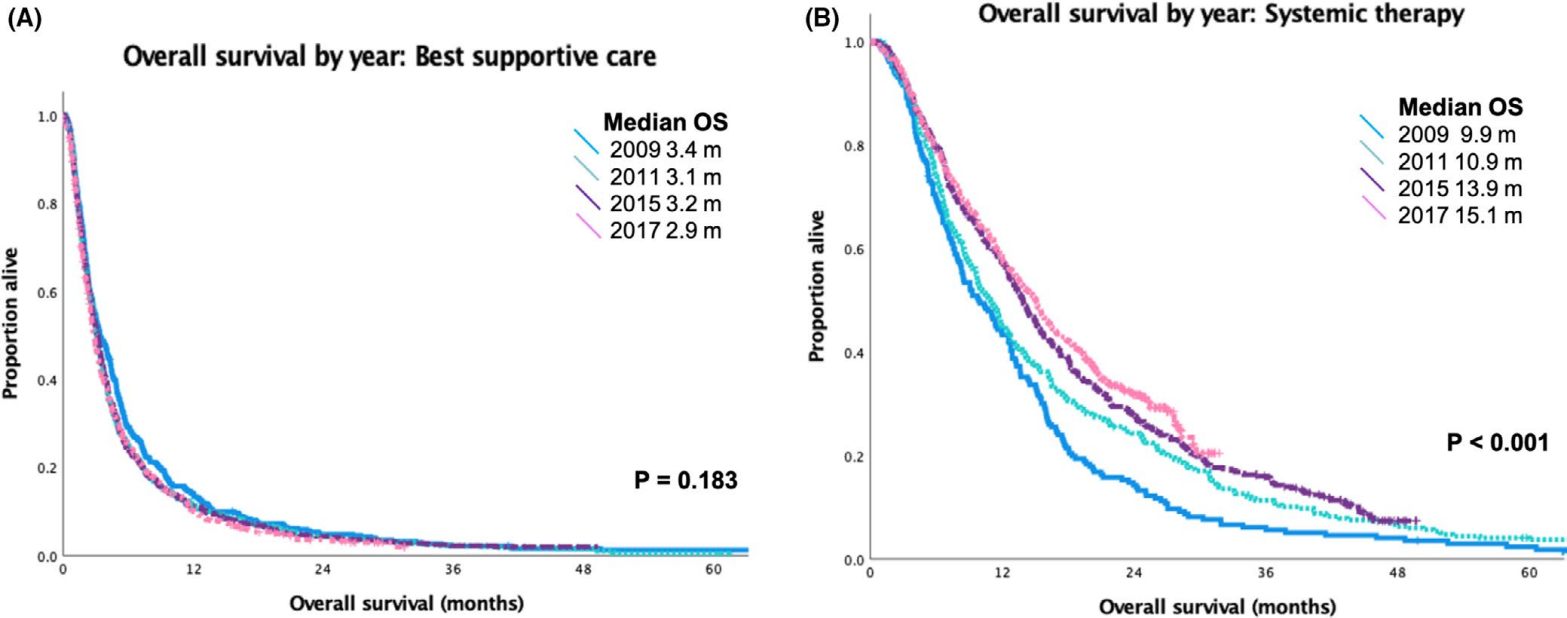
Overall survival (OS) by year for patients diagnosed with stage IV nonsmall‐cell lung cancer receiving (A) best supportive care and (B) treatment with systemic therapy

Median OS (mOS) for patients receiving BSC in the four time cohorts demonstrated no significant difference, ranging from 2.9 to 3.4 months (*p* = 0.16) (Figure 1A). In patients who were treated with systemic therapy, there was a significant increase in the mOS over time, 9.9/10.9/13.9/15.1 months (*p *< 0.001) (Figure 1B). The median OS for all patients (BSC and treated with systemic therapy) in the four time cohorts was 5.1/5.0/5.2/5.3 months (*p *= 0.011).

**FIGURE 2:**
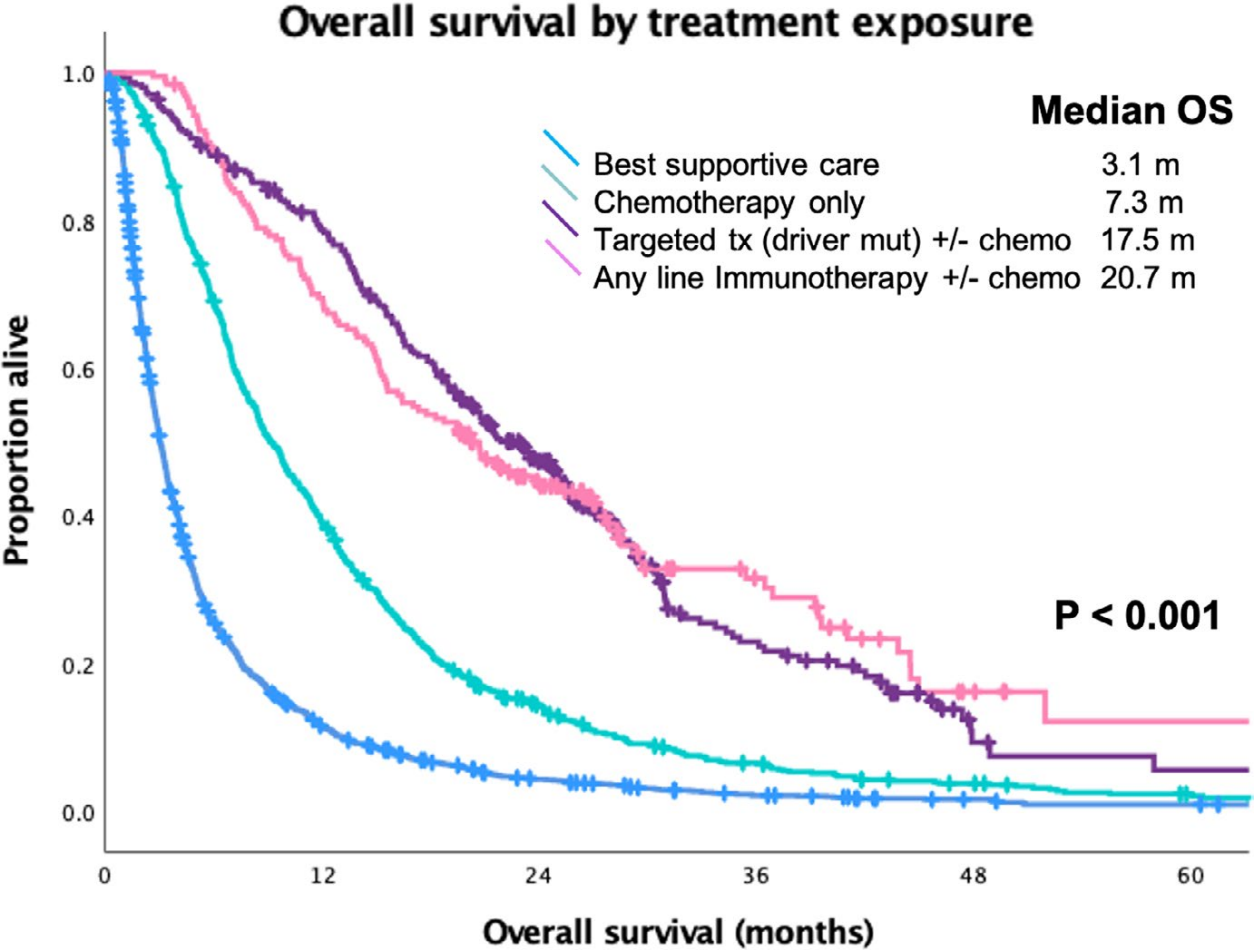
Overall survival (OS) of the whole study population by treatment exposure for patients diagnosed with stage IV nonsmall‐cell lung cancer

OS was calculated using the whole population by treatment exposure during the course of their disease. The median OS for patients receiving BSC was 3.1 months, patients treated with chemotherapy alone 9.2 months, driver mutation receiving targeted therapy (EGFR and ALK) who may have received chemotherapy in another line of treatment 17.5 months, and immunotherapy in any line of treatment who may have received chemotherapy concurrently or sequentially 20.2 months (*p* < 0.001) (Figure 2).

Univariate analysis for factors impacting OS noted that female sex, younger age, nonsquamous histology, ECOG PS 0–1, never smoking, and active systemic treatment was associated with better outcomes (Table 3). On multivariate analysis, the same variables were associated with better outcomes, except older age was more favorable and nonsquamous versus squamous histology was no longer statistically significant. The MVA HR for OS when controlling for other potential factors compared with best supportive care were chemotherapy alone 0.49 (95% CI 0.44–0.53), driver mutation receiving targeted therapy 0.26 (95% CI 0.22–0.30), and immunotherapy in any line of treatment 0.22 (95% CI 0.18–0.27).

**TABLE 3:** Univariate and multivariable analyses of overall survival for patients with advanced nonsmall‐cell lung cancer

	Univariate analysis		Multivariate analysis	
	HR (95% CI)	*p*‐value	HR (95% CI)	*p*‐value
Male versus Female	1.34 (1.25–1.44)	<0.001	1.25 (1.17–1.35)	<0.001
Age (cont., years)	1.01 (1.01–1.01)	<0.001	0.99 (0.99–1.00)	<0.001
Histology				
Nonsquamous	Reference		Reference	
Squamous	1.39 (1.25–1.54)	<0.001	1.05 (0.95–1.17)	0.359
NOS and other	1.46 (1.35–1.59)	<0.001	1.09 (1.00–1.18)	0.04
ECOG at diagnosis				
PS 0–1	Reference		Reference	
PS ≥ 2	2.16 (2.00–2.34)	<0.001	1.75 (1.61–1.90)	<0.001
Unknown	2.11 (1.87–2.37)	<0.001	1.49 (1.32–1.68)	<0.001
Smoking History				
Never	Reference	<0.001	Reference	
Current/Former	1.73 (1.56–1.92)	<0.001	1.14 (1.02–1.28)	0.024
Unknown	2.21 (1.75–2.79)		1.09 (0.86–1.38)	0.478
Treatment				
Best supportive care	Reference		Reference	
Chemotherapy only	0.45 (0.42–0.49)	<0.001	0.49 (0.44–0.53)	<0.001
Driver mutation treated with targeted therapy ± chemotherapy	0.22 (0.19–0.25)	<0.001	0.26 (0.22–0.30)	<0.001
Any line immunotherapy ± chemotherapy	0.21 (0.17–0.25)	<0.001	0.22 (0.18–0.27)	<0.001

Abbreviations: CI, confidence interval; ECOG, Eastern Cooperative Oncology Group; HR, hazard ratio; NOS, not otherwise specified; PS, performance status.

## 4 DISCUSSION

Our retrospective study provides insights into real‐world treatment patterns and survival outcomes for patients diagnosed with advanced NSCLC. Over the four time cohorts, the proportion of patients who received systemic therapy did not change; however, there was the increasing adoption of new therapies that translated into improvements in OS. Examination of survival outcomes by exposure to different systemic therapies clearly showed that targeted therapy and immunotherapy, respectively, were superior to chemotherapy alone. Advances in treatment for advanced NSCLC demonstrate appreciable gains in OS in the real world.

Our study examined four cohorts that reflected an 1‐year period after the introduction of a new therapeutic option in the province of BC. Examination of the patient population over the years demonstrates no clinically significant variations in baseline characteristics. The shift in not otherwise specified (NOS) histology categorization to squamous and nonsquamous from 2011 forward reflects the growing need to differentiate for the purpose of selecting appropriate systemic therapy. The majority of patients was referred with ECOG PS ≥2 at all time points which likely influenced uptake of treatment. Lung cancer screening for early detection was only available in BC through a clinical trial during the period of this study, and consequently, most cases were detected as symptomatic presentation.^13^ The number of patients in 2009 was lower than the other years because the American Joint Committee on Cancer Version 6 was used to stage patients in the registry. As a result, patients with malignant effusions (in version 6 considered T4 vs M1 in version 7) were excluded from the study.^14^ Similarly, patients with an ipsilateral nonprimary lobe nodule were included in 2009 and excluded in subsequent years. Overall, the study population appeared comparable for the evaluation of the impacts of systemic therapy.

The uptake of systemic therapy improved after 2009 but was relatively consistent thereafter. Over the years, >60% of patients received the best supportive care only, which may be a consequence of the high proportion of patients with poor PS. Other population‐based studies have noted similar challenges with systemic therapy delivery in advanced NSCLC patients.^15^, ^16^ With the introduction of molecular testing and the availability of EGFR TKIs and ALK inhibitors, more patients received appropriate targeted therapy over time. The use of second‐line EGFR TKIs was more common in the earlier time cohorts because it was given to an unselected population, based on the data from BR21 with erlotinib demonstrating a survival benefit in second‐ or third‐line patients regardless of EGFR mutation status.^17^ In 2017, the proportion of patients treated with targeted therapy was 34%. This is likely a reflection of higher uptake of targeted therapy among patients with marginal functional status compared with chemotherapy, rather than the true incidence of targetable driver mutations within the population. Immunotherapy was first funded in BC as the second‐line treatment in 2017; however, there was some earlier use of immunotherapy, attributable to delivery on clinical trial for selected fit patients. Utilization in the second‐line setting was 55% of those treated, suggesting relatively prompt incorporation into treatment paradigms. Studies in the United States also note a positive trend for the adoption of immunotherapy.^18^, ^19^

To examine the impact of new therapies on patients, we examined the survival outcomes over time and by treatment exposure. Over the four cohorts, the median OS for patients receiving the best supportive care was 2.9–3.4 months (*p* = 0.163). This suggests that the patient populations are comparable across the four cohorts. Similar to other studies, for patients who were treated with systemic therapy, the median OS improved steadily from baseline 9.9 m to 10.9 m after the introduction of EGFR testing and TKIs, 13.9 m with ALK testing and inhibitors, and 15.1 m with the funding of second‐line immunotherapy.^20^ The apparent benefits of each new therapeutic option may appear more augmented as it also reflects the uptake of the testing and treatment of the prior time cohorts.

Overall survival by treatment exposure highlights the benefits of the introduction of targeted therapy and immunotherapy. In the multivariate analysis controlling for sex, age, histology, ECOG, and smoking status, the reduction in the risk of death compared with best supportive care was HR 0.26 (median OS 17.5 m) for driver mutations receiving targeted therapy. Based on the timing of our study and regulatory approvals in Canada, gefitinib and afatinib were the most commonly used EGFR TKIs for EGFR mutation‐positive patients. The IPASS study enrolled treatment naïve patients with a light‐ or never‐smoking history and adenocarcinoma and had a median OS of 21.6 m with gefitinib.^21^ LUX‐Lung 3 and LUX‐Lung 6 studies had median survivals from first‐line treatment with afatinib of 28.2 and 23.1 m, respectively.^3^ During the study period, crizotinib was funded by the province and in PROFILE 1014, the median OS exceeded 45.8 m at the last analysis.^4^ The majority of the patients in our study had EGFR mutations, and a smaller fraction had ALK fusions, given the timing of the study (second‐line osimertinib was not standardly available) and the real‐world population, the OS aligns with expectations.

The median OS was 20.7 m from diagnosis of metastatic disease for patients who received immunotherapy in any line of therapy, typically a second line or greater. The phase III trials of second‐line nivolumab had a median OS of 12.2 m in advanced nonsquamous and 9.2 m in squamous NSCLC.^10^, ^11^ The second‐line study in advanced NSCLC with PD‐L1 >1% of pembrolizumab at 2 mg/kg and 10 mg/kg every 3 weeks compared with docetaxel had a median OS of 14.9 and 17.3 m from initiation of second‐line therapy.^12^ Our results for the integration of targeted therapy and immunotherapy are congruent with expectations based on clinical trial data and confirm the translation of the evidence to the real‐world population. Although baseline factors still have a significant role in determining prognosis, appropriate systemic therapy is a critical factor influencing outcomes.

The limitations of this study include the retrospective nature, missing data regarding molecular testing rates, referral rates to a medical oncologist, and patient comorbidities, which are important variables when evaluating treatment patterns and uptake of systemic therapy. Potential sources of bias in this study could be improvements in treatment‐related supportive care and stage migration with the greater availability of computed tomography scans and positron emission tomography scans over time. However, their effects are considered to be minimal given that there was no significant difference in overall survival for patients treated with best supportive care by year of diagnosis. A significant confounder is that immunotherapy was funded in the second‐line setting, therefore, introducing a selection bias for those well enough to receive second‐line treatment. Our study strengths include a population‐based cohort subject to patterns of practice consistent with real‐world data through a centralized cancer care program that ensures provincial implementation of guideline changes and recommended therapies. Due to Canada's single‐payer healthcare system, there were no financial barriers in accessing care, ensuring a more realistic portrayal of treatment rates for patients with advanced NSCLC.

In summary, our population‐based study demonstrates a significant improvement in overall survival in advanced NSCLC patients with the implementation of testing for driver mutations and the introduction of targeted therapy and immune checkpoint inhibitors. Our findings in a real‐world population are in line with the results shown in clinical trial populations and demonstrate that it is critical to identify patients appropriately for emerging systemic therapies to provide the best treatment possible.

## CONFLICT OF INTEREST

Dr. Pender reports receiving personal fees from Guardant Health and Bristol Myers Squibb, outside the submitted work; Ms. Leung reports receiving personal fees from Takeda Canada, outside the submitted work; and Dr. Ho reports receiving grants and personal fees from AstraZeneca, EMD Serono, and Roche, and personal fees from Bayer, Bristol Myers Squibb, Eisai, Merck, Novartis, and Takeda Canada, all outside of the submitted work. The remaining authors declare no conflict of interest.

## ETHICAL APPROVAL STATEMENT

This study was approved by the University of British Columbia BC Cancer Research Ethics Board and did not involve direct patient care.

## Data Availability

The data sets used and/or analyzed during the current study are available from the corresponding author upon reasonable request.
